# Cost of bladder cancer in Lebanon before and after the economic collapse: a probabilistic modeling study

**DOI:** 10.1186/s12939-023-01885-8

**Published:** 2023-05-02

**Authors:** Elie Raad, Samar Helou, Karl Hage, Melissa Daou, Elie El Helou

**Affiliations:** 1 Faculty of Medicine, Saint Joseph University of Beirut, Beirut, Lebanon; 2 Global Center for Medical Engineering and Informatics, Osaka University, Osaka, Japan

**Keywords:** Bladder cancer, Cost of illness, Probabilistic model, Out-of-pocket, Economic collapse, Lebanon

## Abstract

**Background and objectives:**

Lebanon has one of the highest incidence rates of bladder cancer (BC) in the world. In 2019, Lebanon’s economy collapsed which majorly impacted healthcare costs and coverage. This study assesses the overall direct costs of urothelial BC in Lebanon, from the perspective of public and private third-party payers (TPP) and households, and evaluates the impact of the economic collapse on these costs.

**Methods:**

This was a quantitative, incidence-based cost-of-illness study, conducted using a macro-costing approach. Costs of medical procedures were obtained from the records of various TPPs and the Ministry of Public Health. We modeled the clinical management processes for each stage of BC, and conducted probabilistic sensitivity analyses to estimate and compare the cost of each stage, pre-and post-collapse, and for each payer category.

**Results:**

Before the collapse, the total annual cost of BC in Lebanon was estimated at LBP 19,676,494,000 (USD 13,117,662). Post-collapse, the total annual cost of BC in Lebanon increased by 768% and was estimated at LBP 170,727,187,000 (USD 7,422,921). TPP payments increased by 61% whereas out-of-pocket (OOP) payments increased by 2,745% resulting in a decrease in TPP coverage to only 17% of total costs.

**Conclusion:**

Our study shows that BC in Lebanon constitutes a significant economic burden costing 0.32% of total health expenditures. The economic collapse induced an increase of 768% in the total annual cost, and a catastrophic increase in OOP payments.

**Supplementary Information:**

The online version contains supplementary material available at 10.1186/s12939-023-01885-8.

## Highlights

Lebanon has one the highest Bladder Cancer (BC) incidence rates. One study examined the cost of BC, from 2008 to 2017 using data from one TPP, and estimated the average annual cost of BC at 3538 USD per patient. These estimates might not reflect the current reality since the 2019–2020 Lebanese economic collapse majorly impacted healthcare utilization, management, and costs. Moreover, the cost of BC from Lebanese households’ perspective remains unknown.

Before the 2019–2020 economic collapse, the total annual cost of BC in Lebanon was estimated at LBP 19,676,494,000 (USD 13,117,662). TPPs covered 89% and 11% was paid OOP. Post-collapse, the total annual cost of BC in LBP increased by 768%. TPP payments increased by 61% whereas OOP payments increased by 2,745%.

This paper sheds light on the catastrophic increase in OOP payments in BC patients post-collapse, informing the decision makers of the urgency to tackle this subject and the crucial need to support households. In addition, due to the devaluation of the LBP, the total cost of BC per patient in USD dropped by 43% post-collapse, which implies a readjustment of international aid allocations, as they can be expected to cover a larger number of patients.

**Supplementary Information:**

The online version contains supplementary material available at 10.1186/s12939-023-01885-8.

## Background

Bladder cancer (BC) is the tenth most frequently diagnosed cancer worldwide, with an adjusted incidence of 572,778 new cases in 2021 [1]. As of 2022, the prevalence stands at 1,720,625 cases over a 5-year period [1]. Several published studies highlight the male predominance of BC with a sex ratio that varies between 6:1 and 2:1 [2,3]. Even though BC incidence and mortality rates in Europe and the United States are among the highest worldwide [1,4], they are significantly increasing in countries undergoing rapid economic development such as South American and Asian countries [4].

Based on an epidemiological study conducted by Lakkis et al. (2018) using data from the Lebanese National Cancer Registry over a 7-year period (2005–2011), BC is the third most common cancer in Lebanon [5]. It accounts for 9% (781 cases/year) of all diagnosed cancer cases [5]. Although not considered an industrialized country, Lebanon has one of the highest estimated age-standardized incidence rates for BC while also having the highest incidence of BC in women in the world (2.8/100,000) [6]. These rates surpass those found in neighboring countries such as Egypt and Tunisia and are comparable to some Western European countries [5].

From an economic standpoint, BC represents a considerable economic burden. It is the most expensive cancer in the elderly population and the ninth most expensive cancer in terms of treatment in the United States [7,8]. In Lebanon, only one retrospective study assessed the cost of BC. This study was conducted between 2008 and 2017, from a private third-party payer (TPP) perspective, and reported an average annual cost of 3,538 USD per BC patient [9]. Even though this study provided important insights into the cost of BC in Lebanon, there is currently no data regarding the cost of BC from the patients’ perspective and public TPPs. Therefore, further cost-of-illness studies are needed to understand the real economic burden of BC in Lebanon. Moreover, since late 2019, Lebanon has been undergoing an economic and financial collapse ranked among the three most severe global collapse episodes since the mid-19th century [10]. Lebanon’s healthcare capacity is assured by public and private hospitals with the latter providing 82% of the services [11]. Unfortunately, private hospitals are owed 1.3 billion USD, and received few to no government funds since the start of the collapse [11]. In addition, the inflation and currency depreciation that ensued led to an astounding increase in healthcare-related costs particularly on Out of Pocket (OOP) payers. Consequently, the cost of BC management in Lebanon has potentially skyrocketed and remains undetermined.

Cost-of-illness studies are among the most common economic studies used by national and international monetary and economic institutions, such as the World Bank, WHO, and the National Institute of Health (NIH) [12,13]. Their main objective is to assess the economic burden that the disease and its complications impose on society in terms of consumption of healthcare resources and lost output [14]. These studies present a great challenge in Lebanon given the difficulty in collecting data due to the complex and fragmented Lebanese health information system; the large differences in health coverage between the public and private sectors; and the disparity in healthcare access between rural and urban areas [15].

Given the high incidence of BC in Lebanon, and the importance of having an up-to-date assessment of its economic burden, this study aims to assess the overall direct costs associated with BC’s diagnosis, treatment, and follow-up. This assessment is conducted from the perspective of various public and private third-party payers, and households’ OOP payments. Furthermore, due to the rapid economic changes that the country has witnessed in the last three years, we also assess the impact of the economic collapse on BC cost, comparing a pre-and a post-collapse scenario.

## Materials and methods

We designed an incidence-based model and conducted multi-way probabilistic sensitivity analyses (multi-way PSAs) to obtain a 1-year estimate of the direct costs of BC in Lebanon, from TPPs’ and households’ perspectives. The model represents real-life pathways of a hypothetical cohort of patients diagnosed with different stages of BC and followed over 5 years.

Since the management and cost of BC were impacted by the economic collapse of 2019–2020, we adjusted the model’s input parameters to simulate pre-collapse and post-collapse scenarios. Moreover, in the post-collapse period, the Lebanese Central Bank (BDL) started subsidizing certain medical treatments to alleviate the medical collapse. Therefore, we also simulated BDL’s contributions during the post-collapse period. Accordingly, 5 simulations were conducted representing the following scenarios:

- Pre-collapse TPP: The direct cost of BC from TPPs’ perspective before mid-2019.
- Pre-collapse OOP: The direct cost of BC from households’ perspective before mid-2019.
- Post-collapse TPP: The direct cost of BC from TPPs’ perspective after mid-2020.
- Post-collapse OOP: The direct cost of BC from households’ perspective after mid-2020.
- Post-collapse BDL: The direct cost of BC from BDL’s perspective after mid-2020.

In addition, one extreme scenario was simulated to analyze the effect of a complete end of BDL’s subsidies on the direct costs for TPP and OOP in the post-collapse period.

Data included in the model and simulations were obtained through reviews of PubMED literature, local databases and registries, and a Delphi process conducted with local experts.

### Model structure

The model was structured as a tree diagram where each branch of the tree represents a real-life patient pathway and accounts for the percentage of BC patients who follow this pathway.

The model starts with a diagnosis of a bladder polyp followed by a timely or late trans-urethral resection of a bladder tumor (TURBT). Patients diagnosed with BC could fall into non-muscle-invasive BC (low, intermediate, and high risk groups), muscle-invasive and metastatic BC. Each group has three possible follow-up pathways: optimal, sub-optimal, or no follow-up.

The guidelines of the European Association of Urology (EAU) were used to model the optimal follow-up pathways. The sub-optimal pathways were modeled based on modifications of the optimal pathway by reducing the number of procedures, adjuvant and neoadjuvant treatments in a realistic way that reflects real-world behavior in Lebanon. Assumptions made for the modeling of sub-optimal pathways are described in detail in the appendix.

**Fig. 1:**
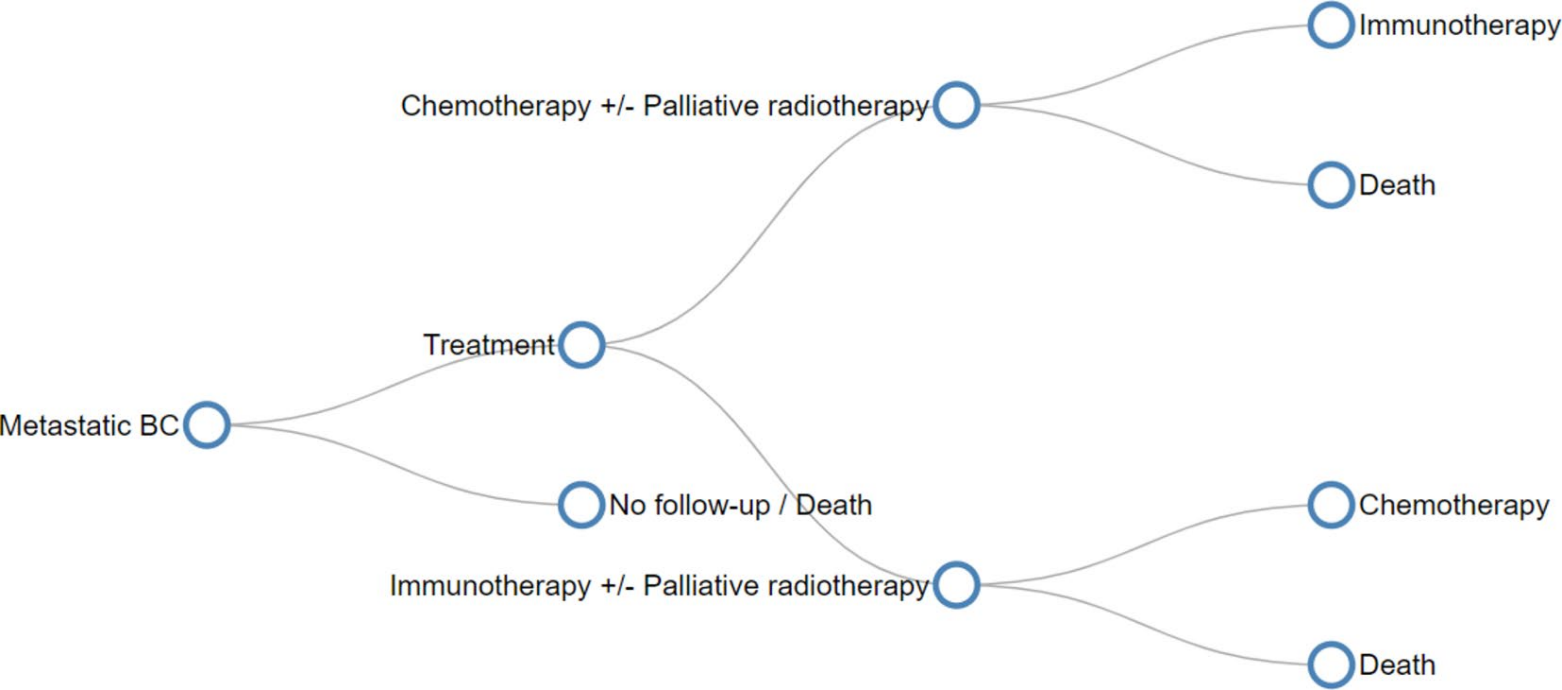
Structure of metastatic BC pathway

Figure 1 shows an example of the structure of metastatic BC pathways. The full model is too large to showcase in a document and is available online at https://usjuroteam.github.io/BC_COI/.

The model was designed using the Decision Tree Software version 5.0 (SpiceLogicTM Inc. Thornhill, Ontario, Canada).

### Model input parameters

#### Probability parameters

Data from the literature and the guidelines of the European Association of Urology (EAU) [16–24] were used to establish the proportions of each BC stage, the probabilities of recurrence and progression, progression-free survival rates, and mortality rates in the optimal follow-up pathways, as well as the complication rates for each treatment procedure. Assumptions were made about the probabilities of the different stages of BC if TURBT was performed late, about recurrence, progression, and mortality rates in the sub-optimal follow-up pathways. These assumptions are fully described in the appendix.

The percentages of patients receiving optimal, sub-optimal, or no follow-up were not available in existing literature or local databases and therefore were estimated based on local experts’ opinions using the conventional Delphi method. This method is suitable in health science studies to develop an expert-based consensus judgment about a given issue [25,26]. We conducted this method with a group of 5 urologists and 5 oncologists who are experts in BC and practicing in different regions of Lebanon.

#### Cost parameters

We adopted a macro-costing approach, from TPPs’ and households’ perspectives. The following TPPs were included: private insurances, the National Social Security Fund (NSSF), the state civil servants’ cooperative, the army, the Internal Security Forces (ISF), and the Ministry of Public Health (MoPH). In the post-collapse period, BDL subsidies for drugs used in BC treatment were included when applicable.

The healthcare procedures included are the following: physician consultation (according to the official tariff of the Lebanese Order of Physicians), urinalysis, urine culture, pelvic ultrasound, CT urogram, thoraco-abdomino-pelvic CT scan with contrast (TAP scan), cystoscopy, transurethral resection of bladder tumor (TURBT), radical cystectomy, intravesical instillation of mitomycin C (MMC), intravesical instillation of Bacille Calmette-Guérin (BCG), different chemotherapy protocols (ddMVAC, Gem-Cisplat, Gem-Carbo, MMC + 5-FU), immunotherapy protocols (pembrolizumab and atezolizumab), curative pelvic radiation therapy, and palliative radiation therapy. Table 1 shows the prices for each of these procedures.

**Table 1:** Costs of procedures (in thousands of LBP)

Procedure	Pre-collapse cost (1,000 LBP)			Post-collapse cost (1,000 LBP)		
	TPP	OOP		TPP	OOP	BDL
Physician consultation	80	20		80	320	-
Urinalysis	5.6	1.4		5.6	46	-
Urine culture	24	6		24	196	-
Pelvic ultrasound	118	29		118	582	-
CT urogram	224	56		224	1,176	-
TAP scan with contrast	224	56		224	3,116	-
MMC instillation	444	111		444	467	5,250
BCG instillation	314	79		314	471	2,800
Radio-Chemo TMT	6,373	12,200		6,373	89,133	9,660
Palliative radiotherapy	2,000	3,000		2,000	32,000	-
Cystoscopy	798	63		1,883	4,762	-
TURBT	1,796	135		4,673	10,290	-
Radical cystectomy	8,579	697		19,667	52,017	-
ddMVAC 4 cycles	7,028	563		7,028	6,189	64,512
Gem-Cisplatin 4 cycles	5,856	427		5,856	6,121	47,600
Gem-Carboplatin 4 cycles	5,279	356		5,279	6,318	38,472
Immunotherapy 1st line	30,548	-		30,548	-	74,455
Immunotherapy 2nd line	100,884	-		100,884	-	1,244,547

*BCG, Bacille Calmette-Guérin; BDL, Banque du Liban; ddMVAC, Dose-Dense Methotrexate, Vinblastine, Adriamycin, Cisplatin; Gem, Gemcitabine; OOP, Out-Of-Pocket; LBP, Lebanese Pound; MMC, Mitomycin C; TAP, Thoraco-Abdomino-Pelvic; TMT, Tri-Modal Therapy; TPP, Third-Party Payers; TURBT, Transurethral Resection of Bladder Tumor*

For each procedure, we collected the direct costs paid by TPPs and households, before and after the economic collapse. TPP and OOP payments were obtained by consulting the coverage databases of the various TPPs, taking into account the proportion of the Lebanese population covered by each TPP [15]. Drug costs were obtained from the Lebanese National Drug Database issued by the Ministry of Public Health [27]. In the post-collapse scenario, BDL subsidies amounted to 93% of drug prices at the time the modeling was performed

### 1-year cost calculation

The cost of each investigation and treatment is calculated by summing the prices of included procedures. Two researchers double-checked the entries to ensure their accuracy.

The cost of BC for a year $$ x$$ is calculated by the following formula:

$$\begin{aligned}& C(x) = I\left( {x - 1} \right) \times C({A_1}) + I\left( {x - 2} \right) \times C({A_2})\\ & + I\left( {x - 3} \right) \times C({A_3}) + I\left( {x - 4} \right) \times C({A_4}) + I\left( {x - 5} \right)\\ & \times C({A_5})\end{aligned}$$

Where C($$ x)$$= total average cost of BC in the year $$ x$$, *I*($$ x)$$= incidence of BC in Lebanon in the year $$ x$$, *I*($$ x-1)$$= incidence of BC in the year prior to $$ x$$, C(A1) = cost in the first year of follow-up, C(A2) = cost in the second year of follow-up, etc.

Assuming that the incidence of BC in Lebanon is stable over the years (equal to 781 per year [5]), we obtain the following:

$$\eqalign{  & I(x - 1 = I\left( {x - 2} \right) = I\left( {x - 3} \right)  \cr   &  = I\left( {x - 4} \right) = I\left( {x - 5} \right) = I(x) = 781 \cr} $$

Which makes:

$$ \begin{array}{l}C\left( x \right) = I\left( x \right)\\\times \left[ {{\rm{C}}\left( {{A_1}} \right) + {\rm{C}}\left( {{A_2}} \right) + {\rm{C}}\left( {{A_3}} \right) + {\rm{C}}\left( {{A_4}} \right) + {\rm{C}}\left( {{A_5}} \right)} \right]\\= I\left( x \right) \times \sum C \left( A \right) = 781 \times \sum C \left( A \right)\end{array} $$

Where $\sum C\left(A\right)$= the overall cost calculated through the model.

The costs obtained are those of 2019 prior to the collapse, and of 2022 after the collapse. No discounts were made as these are the total direct costs of BC for one year. Costs were collected and calculated in Lebanese pounds (LBP), then converted to US dollars (USD) at the rate of USD 1 = LBP 1,500 pre-collapse, and USD 1 = LBP 23,000 post-collapse (exchange rate of BDL at the time of the calculations).

### Probabilistic sensitivity analyses

Multi-way PSAs, in the form of Monte Carlo simulations, were conducted for all scenarios using R version 4.1.1. These PSAs account for the variability and uncertainty in the model’s input parameters as real-life costs and probabilities may vary across institutions, regions, and doctors. It is important to note that prior to conducting the PSAs, the model was internally validated by varying the input parameters and by comparing results of the model in the Decision Tree Software and the one scripted in R-studio.

Each input variable was modeled as a probability distribution taking into account its type (cost parameter versus probability parameter) and its original estimate (the value estimated using the methods described in previous sections). All unit costs were modeled as Gamma distributions with a mean equal to the estimated cost and a standard deviation equal to 20% of the mean. Probability parameters were modeled using either Beta-PERT or Dirichlet distributions depending on the number of branches. For two branches, each branch probability followed a Beta-PERT distribution where the mode was equal to the branch’s estimated probability with a 40% min-max range. For three or more branches, the probabilities were modeled as a Dirichlet distribution i.e., a multivariate beta distribution (MBD) with the input parameters being equal to the original estimates.

Each Monte Carlo simulation consisted of 100,000 runs and resulted in a probability distribution for the total direct cost of BC from which we report the mean cost and its 95% CI.

## Results

Table 2 shows the probability parameters obtained by expert opinions using the conventional Delphi method. In the post-collapse period, expert consensus shows a decrease in the proportion of patients undergoing optimal follow-up in all BC stages and an increase in the proportion of patients not undergoing any treatment or follow-up (4% post-collapse vs. 1.75% pre-collapse).

**Table 2:** Probabilities in pathways based on expert opinions

Questions	Consensus	
	Pre-collapse (%)	Post-collapse (%)
**Pathway of diagnosis of BC**		
1. When patients are diagnosed with a bladder polyp, how many of them will undergo TURBT?	95	90
2. How many patients do nothing?	5	10
a. If they choose to do nothing, how many patients will come back and have a late TURBT?	65	60
b. If they choose to do nothing, how many patients will never come back?	35	40
**Pathway of low-risk BC**		
1. How many patients do the optimal treatment and follow-up?	50	30
2. How many patients do the suboptimal treatment and follow-up?	30	40
3. How many patients do not do treatment and follow-up?	20	30
**Pathway of intermediate-risk BC**		
1. How many patients do the optimal treatment and follow-up?	40	30
2. How many patients do the suboptimal treatment and follow-up?	50	50
3. How many patients do not do treatment and follow-up?	10	20
**Pathway of high-risk BC**		
1. How many patients do the optimal treatment and follow-up?	30	20
2. How many patients do the suboptimal treatment and follow-up?	65	70
3. How many patients do not do treatment and follow-up?	5	10
**Pathway of very high-risk BC**		
1. How many patients do the optimal treatment and follow-up?	30	20
2. How many patients do the suboptimal treatment and follow-up?	65	70
3. How many patients do not do treatment and follow-up?	5	10
**Pathway of invasive risk BC**		
1. How many patients do the optimal treatment and follow-up?	80	60
a. In the optimal scenario, how many do neoadjuvant chemotherapy + radical cystectomy?	80	90
b. In the optimal scenario, how many do TMT?	20	10
2. How many patients do the suboptimal treatment and follow-up?	15	30
3. How many patients do not do treatment and follow-up?	5	10
**Pathway of metastatic risk BC**		
1. How many patients do treatment and follow-up?	90	80
a. How many patients undertake chemotherapy in the first line?	85	85
b. How many patients undertake immunotherapy in the first line?	15	15
2. How many do not do treatment and follow-up?	10	20

*BC, Bladder Cancer; TMT, Tri-Modal Therapy; TURBT, Transurethral Resection of Bladder Tumor.*

By calculating the proportions of pathways ending in death in pre- and post-collapse scenarios, we estimated the overall mortality caused by BC in each setting: the overall mortality before the collapse was 15.9%, and it increased to 17.5% after the economic collapse.

### 1-year direct costs of BC

Table 3 shows the mean annual costs of BC for TPPs, households, and BDL in the pre- and post-collapse periods, along with their 95% CIs.

**Table 3:** 1-year direct costs of BC

	Mean Cost (1,000 LBP)	CI 95% [Min-Max]	Mean cost (USD)	CI 95% [Min-Max]	% of total Cost
**Pre-collapse**					
TPP	17,459,219	(15,036,910 − 20,173,599)	11,639,479	(10,024,607 − 13,449,066)	89%
OOP	2,217,275	(1,761,330-2,791,719)	1,478,183	(1,174,220-1,861,146)	11%
TOTAL	19,676,494		13,117,662		
**Post-collapse**					
TPP	28,181,835	(23,581,845 − 33,430,735)	1,225,297	(1,025,298-1,453,410)	16%
OOP	63,088,851	(51,758,434 − 76,097,529)	2,742,994	(2,250,367-3,308,588)	37%
BDL	79,456,501	(61,271,540 − 104,583,399)	3,454,630	(2,663,980-4,547,104)	47%
TOTAL	170,727,187		7,422,921		

*BDL, Banque du Liban; LBP, Lebanese Pound; OOP,Out-of-Pocket; TPP, Third Party Payer; USD, United States dollar.*

Prior to the economic collapse, the mean total annual cost of BC was LBP 19,676,494,000 (USD 13,117,662) out of which 89% was covered by TPPs and 11% was paid OOP by households.

After the collapse and the rapid devaluation of the LBP, the mean total annual cost of BC became LBP 170,727,187,000 (USD 7,422,921) out of which 16% is covered by TPPs, 37% by households, and 47% by BDL subsidies.

These results show that after the economic collapse, the total annual cost of BC in LBP increased by 768%. TPP payment increased by 61% and OOP payment increased by a staggering 2,745%.

Table 4 shows how each stage of BC contributes to the total cost. After the collapse, the cost contribution of intermediate risk and high risk BC decreased from 25% and 38–24% and 35%, respectively. On the other hand, the cost contribution of muscle-invasive and metastatic BC increased from 24% and 10–25% and 13%, respectively.

**Table 4:** Percentage of total cost for each BC stage

BC stage	Pre-collapse	Post-collapse
Low risk BC	3%	3%
Intermediate risk BC	25%	24%
High risk BC	38%	35%
Muscle-invasive BC	24%	25%
Metastatic BC	10%	13%

*BC, Bladder cancer.*

### Impact of zero subsidies from BDL

In the current economic situation, the Lebanese Central Bank (BDL) is reducing its subsidies on most goods and medications and may end all subsidies in the future. From this perspective, we consider the impact of the extreme scenario where BDL stops all subsidies on BC chemotherapy and immunotherapy. The results of our simulation of this scenario are shown in Table 5.

**Table 5:** Distribution of costs if BDL stops all subsidies

	Mean Cost (1,000 LBP)	CI 95% [Min-Max]	Mean cost (USD)	CI 95% [Min-Max]	% of total Cost	Difference with Realistic scenario in 1,000 LBP (% of change)
TPP	28,181,835	(23,581,845 − 33,430,735)	1,225,297	(1,025,298-1,453,410)	16%	0 (0%)
OOP	142,980,798	(120,264,065–170,769,823)	6,216,556	(5,228,872-7,424,775)	84%	+ 79,891,947 (+ 127%)
BDL	0	0	0	0	0%	
TOTAL	171,162,633		7,441,853			+ 435,446 (+ 0,25%)

*BDL, Banque du Liban; LBP, lebanese pound; OOP, out-of-pocket; TPP, third party payers; USD, United States dollar.*

If BDL ends all subsidies on BC chemotherapy and immunotherapy, the mean annual cost paid by TPP remains the same, whereas the cost paid OOP increases by 127% (LBP 79,891,947,000) and becomes LBP 142,980,798,000 (USD 6,216,556), therefore representing 84% of the total cost.

### Full adherence to guidelines scenario (100% optimal care)

In addition to analyzing realistic scenarios where some BC patients receive suboptimal or no follow-ups, we ran a simulation where all patients receive the recommended treatments and follow-ups, i.e., optimal care. The costs associated with this scenario are shown in Table 6. Before the collapse, the mean total annual cost for the fully adherent treatment of BC on the Lebanese population was estimated at LBP 19,775,539,000 (USD 13,183,692). Compared to the realistic scenario, this cost reflects an increase of 0,5% (99,045,000 LBP).

For TPPs, the mean annual cost was estimated at LBP 17,395,503,000 (USD 11,597,002).

For OOP, the mean annual cost was estimated at LBP 2,380,036,000 (USD 1,586,690) pre-collapse.

**Table 6:** Full adherence to guidelines scenario

	Mean Cost (1,000 LBP)	CI 95% [Min-Max]	Mean cost (USD)	CI 95% [Min-Max]	% of total Cost	Difference with Realistic scenario in 1,000 LBP (% of change)
TPP	17,395,503	(15,043,835 − 20,011,248)	11,597,002	(10,029,223 − 13,340,832)	88%	− 63,716 (− 0,4%)
OOP	2,380,036	(1,925,135-2,940,476)	1,586,690	(1,283,423-1,960,317)	12%	+ 162,761 (+ 7%)
TOTAL	19,775,539		13,183,692			+ 99,045 (+ 0,5%)

LBP, lebanese pound; OOP, out-of-pocket; TPP, third party payers; USD, United States dollar.

## Discussion

To our knowledge, this study is the first to assess the direct cost of BC in Lebanon from the perspective of TPPs and households. Moreover, by comparing the changes in medical service utilization and direct healthcare costs of BC before and after the onset of the 2019 economic collapse, our study was able to determine the impact of this collapse on the cost of BC management from the payers’ perspective.

As previously mentioned, Lebanon is suffering since late 2019 from one of the worst economic and financial crises in history. It is the repercussion of years of post-civil war government corruption, misuse of public savings, and unsustainable monetary and financial strategies [28] The Lebanese central bank (BDL) pegged the Lebanese Lira (LBP) with USD for years, in what was described by many as a Ponzi scheme [29,30]. This has eventually lead to a cataclysmic devaluation of the LBP and a rapidly soaring inflation, estimated to 145% in 2021 – ranking third worldwide [31]. This economic calamity and the LBP devaluation were the driving force behind the devastating changes in treatment costs seen in our study, which are detailed in what follows.

Prior to the economic collapse, annual expenditures on BC in terms of direct costs reached LBP 19,676,494,000 and constituted 0.32% of total health expenditures (LBP 6,238,412,511,000 in 2017) [27]. Post-collapse, the total annual cost of BC in LBP increased by 768% and disproportionately affected households which saw an increase of 2,745% in OOP payments in comparison with a 61% increase for TPPs. The staggering increase in OOP expenses highlights the burden that BC currently inflicts on Lebanese households. Health systems that require lower OOP payments provide better protection for the most penurious patients against catastrophic spending. When OOP spending is < 15% of total health spending, very few households are affected by catastrophic spending [32,33]. This is not the case in the post-collapse situation in Lebanon, where the OOP payment on BC amounts for 37% of the total cost. This leaves the lowest-income households at risk of serious financial disasters and will surely increase inequity in access to care. As the collapse progresses, we get less and less universal medical coverage, which leads to fewer people adhering to the optimal follow-up. We indeed showed that the optimal follow-up does not increase the cost, but it is established that adhering to it decreases the burden of the disease in terms of progression and mortality [34–36]. That’s why, in times of collapse, governments should prioritize the help directed to maintain a universal coverage.

This post-collapse surge in OOP payments accompanies a drop in the Lebanese minimum wage value and purchasing power of the patients, compared to the dollar. The minimum wage being LBP 675,000 was equivalent to nearly USD 450 before the collapse. In the post-collapse period, it has become equivalent to approximately USD 29. Moreover, in its report in September 2021, the United Nations Economic and Social Commission for Western Asia (ESCWA) reports that 74% of the Lebanese population currently lives below the poverty line [37]. This creates a huge gap between post-collapse BC costs and patients’ means.

However, despite the increase of the average total cost after the collapse in LBP, the same cost calculated in USD has decreased. This sets the ground for many disparities in the Lebanese society by creating a financial gap between patients who receive income in LBP and those who receive income in USD, who are a minority. In early 2022, over 90% of the Lebanese population earn their income in LBP only [38,39], and the professions that earn in LBP are mainly the public civil servants, the army, the security forces, and teachers in the public sector [40]. In addition to these groups, the elderly are among the most affected, since BC is most frequent in those over 65 years [41]. The elderly people represent 11% of the Lebanese population (more than any other Arab country). 84% of them do not receive any pension, and if they do it’s in LBP. In mid-2021, 63% reported difficulty in paying health care costs [42].

Thus, for the group of patients earning in fresh USD, the cost of BC has relatively decreased in the post-collapse period even if the patient is not insured. As for those who wish to get private medical insurance, it is now possible for them to pay reduced premiums in liquid USD due to this drop in the costs in all healthcare services. Subsequently, their access to medical services has become easier and more affordable, which further increases the inequality in access to healthcare among the Lebanese population.

In other respects, our results show that the BDL plays an important role in the post-collapse period, given its contribution of 43.1% of the total cost. Indeed, the BDL pays direct subsidies on certain drugs whose cost increased due to the devaluation of the LBP. But this raises the question of whether these subsidies are to be considered a public fund or part of the households’ unattainable savings. This issue is still up for debate, as some consider them equivalent to a loan to the government represented by the Ministry of Public Health and therefore constitute a public funding, while others consider that these subsidies are derived from citizens’ bank deposits, and therefore constitute a form of private funding.

The BDL subsidies are considered unsustainable in the long run which necessitates the examination of an extreme scenario where BDL’s drug subsidies are completely halted. Our simulation of this scenario showed that OOP payments would reach an alarming rate of 84% of the total cost (LBP 142,980,798,000; USD 6,216,556). Although this extreme scenario will result in a drop of optimal care, as already discussed, treatment patterns did not significantly influence the change in total cost.

In terms of medical service utilization, experts’ opinions highlighted a decrease in the proportion of patients fully adhering to follow-up guidelines. This decrease was evident in all stages of BC. For example, the number of patients diagnosed with bladder polyps who did not undergo any further investigation or treatment has more than doubled (1.75% in pre-collapse vs. 4% in post-collapse). Similarly, the proportion of patients seeking medical care in the advanced phase of their disease has almost doubled (3.25% pre-collapse vs. 6% post-collapse). These changes are problematic as delayed diagnosis and management of BC or treatment abstention are associated with a worse prognosis and higher progression and mortality rates [34–36]. This is in concordance with our results, where our model estimated an increase in the mortality rate from 15.9 to 17.5%, which is probably a consequence of decreased optimal care due to the economic collapse.

In the same perspective, with the assumption that all the patients and physicians adhere to the guidelines and follow optimal care patterns before the collapse, the overall cost of BC on the Lebanese population only increased by 0.5%. This means that changes in cost are not attributed to changes in treatment pattern. We did not include this scenario in the post-collapse period as it is counter intuitive to study an optimal scenario in an economic crisis where care is shifting away from being optimal.

As we established that BC constituted a burden on the Lebanese population, it is important to address both its high incidence rates and costs. Given the fact that tobacco smoking is the main risk factor for BC and 42.5% of the Lebanese population older than 15 years consume tobacco [43], it would be possible to reduce BC incidence and costs by effectively implementing anti-smoking policies. For instance, decision makers should enforce the 2011 law No. 174/2011 which regulates smoking in workplaces and public spaces, as well as tobacco manufacturing and advertising [44]. Another measure would be to educate the population and intensify awareness campaigns since only 28% of the Lebanese population is aware of the relationship between tobacco smoking and BC [45].

Although no BC screening tool has yet been adopted, there is emerging evidence in favor of BC screening by home urine dipstick and other biomarkers [46]. Sievert et al. found that BC screening contributes to cost savings and a 50% reduction in disease progression and results in a gain of 3 years of life per 1,000 patients, thereby decreasing the disease burden [47]. Given the high incidence of BC in Lebanon, the cost-effectiveness of BC screening could be the subject of future studies in the Lebanese context.

### Limitations of our study

First, information on service utilization and probabilities needed for the models were not always available or found in the literature. Adopting expert opinions remains a limitation and is subject to recall bias, despite using a validated method.

Second, there are no strict recommendations in the management of BC, whose follow-up may vary according to patient and physician preferences. We tried to overcome this limitation by creating several treatment and follow-up pathways for each stage of BC in a way that reflects clinical reality.

A third limitation is the large and rapid variability of the exchange rate of the LBP to the US Dollar. To address this limitation in post-collapse cost assessments, we used an exchange rate based on the BDL’s platform rate, which is the rate adopted in medical services quotations at the time of the calculations.

A fourth limitation is use of non-Lebanese data for some of the model parameters due to a lack of national data. Even though the incidence rate of BC is relatively high in Lebanon, there is no evidence that the proportion of the different stages of BC is significantly different than the international rates. Nonetheless, it remains preferable to use local datasets for cost of illness studies which underscores the importance of conducting further epidemiologic studies on BC in Lebanon.

Finally, we did not include in our study direct non-healthcare costs (such as transportation) and indirect costs represented by lost productivity and morbidity, which account for 44% of the total cost of BC as found by Gerace et al. [48]. Therefore, our study tends to underestimate the cost of BC by only considering direct healthcare costs. It may be useful to evaluate these costs in future studies to obtain a more accurate estimation of the real economic burden that BC imposes on the Lebanese population.

## Conclusion

Our study is the first of its kind to address the issue of cost of illness variation in the context of an economic collapse, taking into consideration public and private third-party payers and out-of-pocket payments.

Our study shows that bladder cancer in Lebanon constitutes a significant economic burden on the health care system, with an annual cost of LBP 19,676,494,000 (USD 13,117,662) prior to the economic collapse.

We also showed that the economic collapse induced an increase of 768% in the total annual cost (LBP 170,727,187,000; USD 7,422,921), an increase of 2,745% in the out-of-pocket payment, with a parallel decrease in the use of optimal medical services, and increased overall mortality rate.

## Electronic supplementary material

Below is the link to the electronic supplementary material.

Supplementary Material 1

## Data Availability

The model structure is available publicly at https://usjuroteam.github.io/BC_COI/. The code used to run the simulations and the probabilistic sensitivity analysis will be made available to non-profit parties and only for health and medical research. Requests for code should be made to Dr. Elie El Helou and accompanied with a research proposal, approved by an independent review committee. The authors reserve the right to refuse sharing of data in the face of potential adversarial conflicts of interest.

## List of abbreviations

BC: bladder cancer
BCG: bacille Calmette-Guérin
BDL: Banque du Liban
ddMVAC: dose-dense Methotrexate, Vinblastine, Adriamycin, Cisplatin
Gem: Gemcitabine
OOP: out-of-pocket
LBP: Lebanese pound
MMC: mitomycin C
TAP: thoraco-abdomino-pelvic
TMT: tri-modal therapy
TPP: third-party payers
TURBT: transurethral resection of bladder tumor
WHO: world health organization

## References

[1] Richters A, Aben KKH, Kiemeney LALM (2020). The global burden of urinary bladder cancer: an update. World J Urol.

[2] Ferlay J, Soerjomataram I, Dikshit R (2015). Cancer incidence and mortality worldwide: sources, methods and major patterns in GLOBOCAN 2012. Int J Cancer.

[3] Donsky H, Coyle S, Scosyrev E, Messing EM (2014). Sex differences in incidence and mortality of bladder and kidney cancers: national estimates from 49 countries. Urol Oncol.

[4] Antoni S, Ferlay J, Soerjomataram I, Znaor A, Jemal A, Bray F (2017). Bladder Cancer incidence and mortality: A global overview and recent Trends. Eur Urol.

[5] Lakkis NA, Adib SM, Hamadeh GN, El-Jarrah RT, Osman MH (2018). Bladder Cancer in Lebanon: incidence and comparison to Regional and Western Countries. Cancer Control J Moffitt Cancer Cent.

[6] Bray F, Ferlay J, Soerjomataram I, Siegel RL, Torre LA, Jemal A (2018). Global cancer statistics 2018: GLOBOCAN estimates of incidence and mortality worldwide for 36 cancers in 185 countries. CA Cancer J Clin.

[7] Riley GF, Potosky AL, Lubitz JD, Kessler LG (1995). Medicare payments from diagnosis to death for elderly cancer patients by stage at diagnosis. Med Care.

[8] Mariotto AB, Robin Yabroff K, Shao Y, Feuer EJ, Brown ML (2011). Projections of the cost of Cancer Care in the United States: 2010–2020. JNCI J Natl Cancer Inst.

[9] Mesmar A, Sabbagh R, Maskineh C, Hashem GE, Becker RV, PCN87 REAL-WORLD COSTS OF BLADDER CANCER TREATMENT IN LEBANON USING A PRIVATE PAYER DATASET (2019). Value Health.

[10] Monitor LE. Spring 2021: Lebanon Sinking (to the Top 3). World Bank. Accessed July 31, 2022. https://www.worldbank.org/en/country/lebanon/publication/lebanon-economic-monitor-spring-2021-lebanon-sinking-to-the-top-3

[11] Devi S (2020). Economic crisis hits lebanese health care. Lancet Lond Engl.

[12] Byford S, Torgerson DJ, Raftery J (2000). Economic note: cost of illness studies. BMJ.

[13] Bloom BS, de Pouvourville N, Straus WL (2003). Cost of illness of Alzheimer’s disease: how useful are current estimates?. Gerontologist.

[14] Jo C (2014). Cost-of-illness studies: concepts, scopes, and methods. Clin Mol Hepatol.

[15] hrh. Country profile. World Health Organization - Regional Office for the Eastern Mediterranean. Accessed July 31., 2022. http://www.emro.who.int/human-resources-observatory/countries/country-profile.html

[16] Koie T, Ohyama C, Yamamoto H (2015). Differences in the recurrence pattern after neoadjuvant chemotherapy compared to surgery alone in patients with muscle-invasive bladder cancer. Med Oncol Northwood Lond Engl.

[17] EAU Guidelines on MIBC - Uroweb. Uroweb - European Association of Urology. Accessed August 23., 2022. https://uroweb.org/guidelines/muscle-invasive-and-metastatic-bladder-cancer

[18] Dogliotti L, Cartenì G, Siena S (2007). Gemcitabine plus cisplatin versus gemcitabine plus carboplatin as first-line chemotherapy in advanced transitional cell carcinoma of the urothelium: results of a randomized phase 2 trial. Eur Urol.

[19] Balar AV, Castellano D, O’Donnell PH (2017). First-line pembrolizumab in cisplatin-ineligible patients with locally advanced and unresectable or metastatic urothelial cancer (KEYNOTE-052): a multicentre, single-arm, phase 2 study. Lancet Oncol.

[20] EAU Guidelines on Non-muscle-invasive Bladder Cancer - Uroweb. Uroweb - European Association of Urology. Accessed August 23., 2022. https://uroweb.org/guidelines/non-muscle-invasive-bladder-cancer

[21] von der Maase H, Sengelov L, Roberts JT (2005). Long-term survival results of a randomized trial comparing gemcitabine plus cisplatin, with methotrexate, vinblastine, doxorubicin, plus cisplatin in patients with bladder cancer. J Clin Oncol Off J Am Soc Clin Oncol.

[22] Bellmunt J, de Wit R, Vaughn DJ (2017). Pembrolizumab as Second-Line Therapy for Advanced Urothelial Carcinoma. N Engl J Med.

[23] Millán-Rodríguez F, Chéchile-Toniolo G, Salvador-Bayarri J, Palou J, Algaba F, Vicente-Rodríguez J (2000). Primary superficial bladder cancer risk groups according to progression, mortality and recurrence. J Urol.

[24] James ND, Hussain SA, Hall E (2012). Radiotherapy with or without chemotherapy in muscle-invasive bladder cancer. N Engl J Med.

[25] Niederberger M, Spranger J (2020). Delphi technique in Health Sciences: a map. Front Public Health.

[26] de Villiers MR, de Villiers PJT, Kent AP (2005). The Delphi technique in health sciences education research. Med Teach.

[27] moph. Accessed July 31, 2022. http://www.moph.gov.lb

[28] Reuters F. Just how bad is Lebanon’s economic crisis? *Reuters*. https://www.reuters.com/world/middle-east/just-how-bad-is-lebanons-economic-crisis-2022-09-14/. Published September 14, 2022. Accessed April 1, 2023.

[29] Lebanon. Why the country is in crisis. BBC News. https://www.bbc.com/news/world-middle-east-53390108. Published July 17, 2020. Accessed April 1, 2023.

[30] Lebanon’s Ponzi Finance Scheme Has Caused Unprecedented Social and Economic Pain to the Lebanese People. World Bank. Accessed April 1., 2023. https://www.worldbank.org/en/news/press-release/2022/08/02/lebanon-s-ponzi-finance-scheme-has-caused-unprecedented-social-and-economic-pain-to-the-lebanese-people

[31] Lebanon’s Crisis: Great Denial in the Deliberate Depression. World Bank. Accessed April 1., 2023. https://www.worldbank.org/en/news/press-release/2022/01/24/lebanon-s-crisis-great-denial-in-the-deliberate-depression

[32] World Health Organization. Designing Health Financing Systems to Reduce Catastrophic Health Expenditure. World Health Organization. 2005. Accessed July 31, 2022. https://apps.who.int/iris/handle/10665/70005

[33] World Health Organization. Distribution of Health Payments and Catastrophic Expenditures Methodology. World Health Organization. 2005. Accessed July 31, 2022. https://apps.who.int/iris/handle/10665/69030

[34] Hollenbeck BK, Dunn RL, Ye Z (2010). Delays in diagnosis and bladder cancer mortality. Cancer.

[35] Martini A, Sfakianos JP, Renström-Koskela L (2020). The natural history of untreated muscle-invasive bladder cancer. BJU Int.

[36] Fahmy NM, Mahmud S, Aprikian AG (2006). Delay in the surgical treatment of bladder cancer and survival: systematic review of the literature. Eur Urol.

[37] ESCWA warns: Three-quarters of Lebanon’s residents plunge into poverty. ESCWA. Accessed July 31., 2022. http://www.unescwa.org/news/escwa-warns-three-quarters-lebanon%E2%80%99s-residents-plunge-poverty

[38] Lebanon follow-up Labour Force Survey January. 2022. Published June 14, 2022. Accessed April 1, 2023. http://www.ilo.org/beirut/publications/WCMS_848353/lang--en/index.htm

[39] Central Administration of Statistics - Lebanon Follow-up Labour Force Survey January. 2022. Accessed March 26, 2023. http://www.cas.gov.lb/index.php/latest-news-en/201-labour-force

[40] Writer JEDS. “Fresh Dollars”: A New Tool for Exploitation – Watchdogs Gazette. Published June 17, 2021. Accessed March 26, 2023. https://watchdogsgazette.com/affairs/fresh-dollars-a-new-tool-for-exploitation/

[41] Siegel RL, Miller KD, Jemal A, Cancer statistics. 2019. CA Cancer J Clin. 2019;69(1):7–34. doi:10.3322/caac.21551 10.3322/caac.21551 30620402

[42] Launching the National Strategy for Older Persons in Lebanon 2020–2030. United Nations Economic and Social Commission for Western Asia. Published June 9, 2021. Accessed March 26., 2023. https://archive.unescwa.org/events/launching-national-strategy-older-persons-lebanon-2020-2030

[43] World Health Organization. WHO Global Report on Trends in Prevalence of Tobacco Smoking 2000–2025, Third Edition. World Health Organization. 2019. Accessed August 24, 2022. https://www.who.int/publications/i/item/who-global-report-on-trends-in-prevalence-of-tobacco-use-2000-2025-third-edition

[44] Lebanon Details | Tobacco Control Laws. Accessed July 31., 2022. https://www.tobaccocontrollaws.org/legislation/country/lebanon/summary

[45] Souaid T, Hindy JR, Eid R, Kourie HR, Kattan J (2018). Bladder cancer knowledge in the lebanese population: when ignorance could be harmful. Bull Cancer (Paris).

[46] Shahait M, Bulbul M. Bladder Cancer Screening in Lebanese Population: There is Nothing more Unequal than the Equal Treatment of Unequal People.Bladder Cancer Amst Neth. 2(4):467–468. 10.3233/BLC-160074 10.3233/BLC-160074 PMC5181656 28035328

[47] Sievert KD, Amend B, Nagele U (2009). Economic aspects of bladder cancer: what are the benefits and costs?. World J Urol.

[48] Gerace C, Montorsi F, Tambaro R, et al. Cost of illness of urothelial bladder cancer in Italy. Clin Outcomes Res CEOR. 2017;9:433–442. 10.2147/CEOR.S135065. 10.2147/CEOR.S135065 PMC5533568 28769578

