# Supplementary material for: Cost of bladder cancer in Lebanon before and after the economic collapse: a probabilistic modeling study
Source: Int J Equity Health. 2023 May 2;22:77. doi: 10.1186/s12939-023-01885-8 (PMC10152790; doi:10.1186/s12939-023-01885-8)
Supplement: Supplementary file 1 — Supplementary Material 1 [file 12939_2023_1885_MOESM1_ESM.docx]

**Online supplementary appendix**

**1- Interactive model:** <https://usjuroteam.github.io/BC_COI/>

**2- Assumptions for the model’s structure**

- For patients who have undergone delayed TURBT: half of the proportion corresponding to each risk group is added to the higher group, considering that late presentation increases the risk of progression
- The frequency of procedures in the suboptimal follow-up are similar to those in the optimal lower-stage follow-up, without early postoperative intravesical instillation of chemotherapy if indicated.
- The number of procedures in the suboptimal follow-up for low-risk BC patients is half the number of procedures in the optimal follow-up for the low-risk BC patients.
- The suboptimal follow-up of the low-risk BC is equivalent in number and frequency of procedures to half the optimal follow-up of the same stage.
- Suboptimal scenarios had less neo-adjuvant and adjuvant treatments (no neo-adjuvant chemotherapy, less stringent intra-vesical instillations)
- Recurrence and progression rates in the suboptimal scenario of a stage are equivalent to the average of the rates in the optimal scenarios of the same stage and the higher stage.
- The cost of the "death" arm in each BC stage is equivalent to half the arm of patients who remain alive at the end of the 5-year follow-up, assuming that death occurs when the patient has undergone half the procedures.
- The cost of the "no treatment" arm of a BC stage is equal to the cost of the higher stage, assuming that the patient will return later in a more advanced stage.
- If present, progression occurs at the end of the first year of follow-up for all stages.
- After 2 recurrences under intravesical chemotherapy instillation in intermediate-risk BC, there is a switch to intravesical BCG protocol.
- The cost of procedures complications is 30% of the total direct cost, as found by Avritscher et al^1^.
- Because progression and mortality rates for metastatic BC receiving a first line of chemotherapy are missing, we assumed they are equal to those for the first line of immunotherapy.

1. Avritscher EBC, Cooksley CD, Grossman HB, et al. Clinical model of lifetime cost of treating bladder cancer and associated complications. *Urology*. 2006;68(3):549-553. doi:10.1016/j.urology.2006.03.062
